# Characterisation of Bacterial Isolates from Infected Post-Operative Patients in a Malaysian Tertiary Heart Care Centre

**DOI:** 10.3390/ijerph18189828

**Published:** 2021-09-17

**Authors:** Yi Keng Yong, Nicole Ce Mun Wen, Genieve Ee Chia Yeo, Zhi Xin Chew, Li Li Chan, Noor Zaitulakma Md Zain, Dinesh Kumar Chellappan, Yun Khoon Liew

**Affiliations:** 1 School of Health Science, International Medical University, Kuala Lumpur 57000, Malaysia; 00000023095@student.imu.edu.my (Y.K.Y.); NICOLE.WENCE@studentimuedu.onmicrosoft.com (N.C.M.W.); GENIEVE.YEO@student.imu.edu.my (G.E.C.Y.); Chew.zhixin@student.imu.edu.my (Z.X.C.); 2 School of Medicine, International Medical University, Kuala Lumpur 57000, Malaysia; lili_chan@imu.edu.my; 3 Institut Jantung Negara, Kuala Lumpur 50400, Malaysia; akma@ijn.com.my; 4 School of Pharmacy, International Medical University, Kuala Lumpur 57000, Malaysia; Dinesh_Kumar@imu.edu.my

**Keywords:** postoperative patients, bacterial infection, ESBL genes

## Abstract

Several bacterial species cause post-operative infections, which has been a critical health concern among hospital patients. Our study in this direction is a much-needed exploratory study that was carried out at the National Heart Institute (IJN) of Malaysia to examine the virulence properties of causative bacteria obtained from postoperative patients. The bacterial isolates and data were provided by the IJN. Antibiotic resistance gene patterns, and the ability to form biofilm were investigated for 127 isolates. *Klebsiella pneumoniae* (36.2%) was the most common isolate collected, which was followed by *Pseudomonas aeruginosa* (26%), *Staphylococcus aureus* (23.6%), *Streptococcus* spp. (8.7%) and *Acinetobacter baumannii* (5.5%). There were 49 isolates that showed the presence of multidrug resistance genes. The *mecA* gene was surprisingly found in methicillin-susceptible *S. aureus* (MSSA), which also carried the *ermA* gene from those erythromycin-susceptible strains. The phenotypic antibiotic resistance profiles varied greatly between isolates. Findings from the biofilm assay revealed that 44 of the 127 isolates demonstrated the ability to produce biofilms. Our findings provide insights into the possibility of some of these bacteria surviving under antibiotic stress, and some antibiotic resistance genes being silenced.

## 1. Introduction

Post-operative infections constitute a major problem among post-surgical patients, and they are one of the most frequently reported healthcare-associated infections all over the world. More than 30% of health-acquired infections are post-operative infections [1]. Most of the infections are usually caused by endogenous infections, which are derived from microorganisms that are already present in the patient’s body [2,3]. It is well-known that distinct microbiota compositions are observed in different skin sites in humans [4,5]. However, the causative agents may also originate from exogenous factors such as unclean operating rooms and infected surgical instruments [3]. Unsurprisingly the isolated causative agents (either endogenous or exogenous microorganisms) were mostly reported with bacteria, such as *Staphylococcus aureus, Klebsiella pneumoniae,* coagulase-negative staphylococci and *Enterococcus* species [6,7,8].

Post-operative infections after a heart surgery particularly, deep sternal site infections and mediastinitis, increases the rate of morbidity, mortality, and cost. The mediastinitis treatment requires more than 2 weeks additional hospital stay, as reported by Hollenbeak et al. [9]. The proportion of cardiovascular patients at high risk for post-operative infection is increasing due to a growing number of elderly patients who undergo cardiac surgery re-operation. Staphylococci is the predominant pathogen among Gram-positive bacteria isolated in patients with infection at the venous graft harvesting sites after a coronary artery bypass graft and also in patients with mediastinitis [10]. These pathogens play an important role in intraoperative wound contamination by infecting the sternotomy wounds during the long time intervals in open-heart surgery. Consequently, meticulous care and excessive medical supplies such as local blood supply, nutrition, and immunological support services are needed.

As might be expected, the severity of postoperative infections is always associated with prolonged use of invasive medical devices, as well as the long-term hospitalisations in intensive care units (ICU) and hospital wards [11]. In addition, the prolonged use and administration of antibiotics with inappropriate dosing have also resulted in developing resistance towards drugs [12,13,14]. Several studies conducted in certain countries have found the issue of improper use of antibiotics, which is prevalent among healthcare institutions [15,16,17]. All these factors could potentially contribute to the emergence of resistant bacterial strains. The problem of bacterial resistance towards multiple drugs was detected from the clinical isolates, where it comes to the paradigm of intraspecies or interspecies dissemination of antibiotic resistance genes. According to the Centers for Disease Control (CDC), a multidrug-resistant organism is defined as a microorganism that is resistant to one or more classes of antimicrobial agents [18]. Noordin et al. collected 318 MRSA isolates from various hospitals in Malaysia and reported that a number of strains from the total isolates were resistant to gentamicin, ciprofloxacin and erythromycin [19]. Moreover, in another study from Malaysia, researchers revealed the emergence of Gram-negative bacterial isolates that were resistant to multiple classes of antibiotics. For instance, *Acinetobacter baumannii* strains that were observed in a tertiary-care centre in Terengganu (Malaysia), were resistant to more than three types of antibiotic classes and a total of 72.2% were multidrug-resistant. A study conducted by Low et al., found that 39 isolates of *K. pneumoniae* were classified as multidrug-resistant and most of them showed high resistance towards polymyxin [20,21]. Furthermore, a study conducted in Malaysia showed that three multidrug-resistant strains of *Pseudomonas aeruginosa* possessing MBL genes (*bla*_IMP_, *bla*_VIM_ and *bla*_NDM_) showed multidrug resistance to carbapenem, aminoglycosides, cephalosporin, fluoroquinolone and β- lactamase inhibitor (ticarcillin-clavulanic acid) [22].

Biofilm-producing isolates have been a challenging problem in post-operative infections. During hospitalization, patients are exposed to a pathogen-rich environment, thereby increasing the risk of infections. Healthcare workers should raise more awareness on biofilm-producing isolates to minimise the risk of postoperative infections. This is crucial because biofilm-producing strains of bacteria are more problematic than non-biofilm-producing bacteria, due to the ability of the former to secrete extracellular polymeric substances (EPSs) [23]. These EPSs could protect the bacteria by enabling them to resist the attack of antibiotics and host defences [24]. There are several notable reports where the findings have demonstrated that biofilm producers may increase their resistance towards routine surgical preparation protocols such as skin preparation and antisepsis procedures [25,26,27]. In a study conducted by Howard et al., biofilm producer strains of *A. baumannii* were identified as a threat to cause postoperative infections due to their ability to become an opportunistic nosocomial pathogen [28]. Additionally, there were several studies indicating that the majority of the bacteria in biofilms were detected from the genera of *Staphylococcus* and *Acinetobacter* and they were considered as the most common biofilm producers [29,30,31]. A number of studies conducted in Malaysian hospitals also identified that biofilm-forming bacteria were associated with several problems during the treatment of extremely drug-resistant *P. aeruginosa* infections [32,33,34]. Consequently, the increased resistance of biofilm-associated bacterial strains towards cephalosporins and carbapenem could lead physicians to depend on colistin. This drug has been the last-resort antibiotic, which was widely used previously but discontinued in the 1980s as it caused kidney and neurological problems [35,36]. Vuotto et al. also found that the biofilm-forming *K. pneumoniae* strains isolated from medical devices had developed resistance towards amikacin, ciprofloxacin and piperacillin [37].

Despite having numerous investigators who have studied a range of bacterial isolates collected from different healthcare settings in Malaysia, the availability of general data on the characteristics of causative agents with regard to cardiovascular diseases is still meagre. To address these gaps, this study was conducted to explore the characteristics of *S. aureus, K. pneumoniae, A. baumannii, P. aeruginosa* and *Streptococcus* spp. that were isolated from the National Referral Centre for Cardiovascular Disease. The study primarily was focused on profiling the phenotypic and genotypic antimicrobial resistance in such pathogens. Moreover, we further examined the ability of these organisms to form biofilms. The findings from this study may help understand the resistance traits of the above-mentioned bacteria in greater detail. This may further assist in strategizing the postoperative infectious disease management plan for biofilm-related infections and multiple drug resistant bacterial isolates.

## 2. Materials and Methods

### 2.1. Bacterial Strains

A total of 30 *S. aureus,* 46 *K. pneumoniae,* 7 *A. baumannii,* 33 *P. aeruginosa* and 11 *Streptococcus* isolates were kindly provided by the National Heart Institute (IJN) of Malaysia at Kuala Lumpur. These bacterial strains were sampled from blood, wound and respiratory specimens of postoperative patients with cardiovascular diseases. All the pure bacterial cultures were stored at −70 °C in 20% glycerol after being allowed to grow at 37 °C in brain heart infusion (BHI) broth for 24 h.

### 2.2. Ethics

This study was approved by the respective research ethics board, and committees of IJN as each of these isolates was accompanied by patient data that included age, gender, hospital/community acquisition, and the infection source. In addition, the IJN had also provided antimicrobial susceptibility testing provisions for up to 35 antimicrobial agents. All authors ensured that every data, such as the patient profile and medical records, were strictly used only for research purposes, and strict confidentiality was maintained.

### 2.3. DNA Extraction

DNA was extracted from all the isolates with Presto^TM^ Mini gDNA Bacteria Kit (Geneaid Biotech Ltd., New Taipei City, Taiwan) following the manufacturer’s instructions. The quantity and quality of the extracted DNA were determined by Tecan NanoQuant plate infinite F200 (Tecan Group Ltd., Männedorf, Switzerland).

### 2.4. Polymerase Chain Reaction Amplification (PCR) of Resistance Genes

PCR analyses were performed by using an exTEN 2× PCR Master Mix (Axil Scientific Pte Ltd., Singapore) with its PCR primers and programme to screen the antibiotic resistance genes, as shown in Table 1. The PCR products were separated and analysed on a 1.4% agarose gel electrophoresis that was stained with Midori Green Advance (ca. 0.8%) (Nippon Genetics Europe, Düren, Germany). *K. pneumoniae* ATCC BAA-1705, *K. pneumoniae* M48000, *S. aureus* ATCC 4330, and *Enterococcus faecalis* ATCC 51299 were used as control strains for genotypic screening of certain antibiotic resistance genes. Clinical resistant bacterial isolates that harboured the genetic determinants of antibiotic resistance were used as positive controls if there were no reference strains that served as control.

### 2.5. Biofilm Formation Assay

The ability of all the 127 bacterial isolates to form biofilms was evaluated by the method described by Stepanović et al. [53]. The method essentially utilized crystal violet to identify those bacteria, which attached firmly onto the polystyrene surface of a 96-well flat-bottomed microplate. Briefly, the overnight-maintained bacterial culture was diluted with TBS, which contained 1% glucose with a factor of 1:100. All the diluted suspensions (200 μL) were incubated in a 96-well microplate for 24 h at 37 °C without shaking. After that, the contents of each well were washed 3 times with water under vigorous shaking. The adherent bacteria were fixed with 200 μL of 99% methanol for 15 min. Subsequently, the microplates were emptied and were left to dry. The adherent bacteria in each well were then stained with 0.2 mL of crystal violet (2%) for 5 min followed by rinsing with water prior to air-drying. After that, 160 μL of 33% glacial acetic acid was added into each well to solubilize the adherent bacteria. The OD_570_ of each well was determined by a Spectramax M3 spectrophotometer (Molecular Devices, USA). *S. aureus* ATCC 43300 was used as a positive control, whereas negative control wells contained the TBS medium only. The cut-off OD (OD_C_) was expressed as 3 standard deviations above the mean OD_570_ of the negative control. The biofilm producers were categorized as below:

OD ≤ ODC     non-adherent

OD_C_ < OD ≤ 2 × OD_C_     weakly adherent

2 × OD_C_ < OD≤ 4 × OD_C_     moderately adherent

4 × OD_C_ < OD     strongly adherent

## 3. Results

### 3.1. Clinical Samples Detail

All the isolates were collected from postoperative infected patients (69.3% males and 30.7% females) in clinical wards of the IJN between March 2019 and June 2019. The average ages of patients were between 61 and 70 years (range of 1–88 years). Overall, 41 of the total 127 bacterial isolates contained Gram-positive bacteria, and the remaining contained Gram-negative organisms. Few isolates contained Gram-positive bacteria such as *Streptococcus* species, which were frequently identified as *S. dysgalactiae* subspecies *equisimillis* (SDSE) and *S. agalactiae.* Majority of the isolates were found to have originated from blood specimens (60.0%) followed by tracheal aspirates 21.3%; pus/wounds, 17.3%; tissue, 2.4%; bronchial, 1.6%; urine, 0.79% and sputum, 0.79% as shown in Table 2. Notably, it was observed that the isolated *P. aeruginosa* were mostly from respiratory tract samples compared to wounds. There were 70% of the respiratory tract samples identified as *P. aeruginosa*.

### 3.2. Antibiotic Resistance Characteristics of Clinical Isolates

According to the resistance genotype, it was interesting to note that 47.3% of MSSA strains, which supposedly do not harbour the *mecA* gene, were found to co-carry the antibiotic resistance genes, *mecA* and *ermA.* Meanwhile, there was no *ermB* gene detected in MSSA. In this study, both *ermC* and *tetM* genes were also detected in the MSSA, and there were 68.4% of MSSA carrying the *aacA-aphD* gene. Unsurprisingly, the *mecA* gene was detected in all MRSA isolates and these isolates were resistant to oxacillin, which also co-carried *ermC* genes, *tetM*, as well as *aacA-aphD* (36.4%). None of the *S. aureus* strains were detected with *mcr-1, mef(A), mef(E), msrA, tetK, vanA* and *vanB* genes. In contrast, there was one isolate of *K. pneumoniae,* which was found to carry the *mcr-1* gene among all the isolated bacteria. The co-carriage of *bla*_CTX-M_ and *bla*_SHV_ genes was also detected in all *K. pneumoniae* isolates but with the absence of *bla*_KPC-2_, *bla*_IMP_, *bla*_SPM-1_, *bla*_OXA-24_ genes.

As listed in Table 3 and Table S1, 28.6% of *A. baumannii* isolates harbored the *pmrA* and *bla*_GES_ genes, respectively, while six of the seven *A. baumannii* isolates carried the *bla*_SHV_ gene. The presence of the *pmrA* genes was rare in these clinical isolates. Similarly, the *mcr-1, bla*_SPM-1_, *bla*_OXA-24_ and *bla*_OXA-58_ genes were not detected in any of the seven isolates of *A. baumannii.* Among the ESBL genes, *bla*_CTX-M_ was detected in all the *P. aeruginosa* isolates and the *bla*_SHV_ gene was found in 93.9% of the isolates. Surprisingly, more than half of *P. aeruginosa* isolates showed the presence of the *bla*_OXA-23_ gene. All the *P. aeruginosa* isolates were *bla*_OXA-24_ and *bla*_OXA-58_ negative. Similarly, both the *bla*_OXA-24_ and *bla*_OXA-58_ genes were not detected in *A. baumannii* isolates as well. However, a low prevalence of the *bla*_OXA-58_ gene was observed in 6.5% of *K. pneumoniae* isolates. It was observed that 45.5% of *Streptococcus* spp. isolates carried the *ermB* gene but not *ermA* and *ermC* genes. Both the *tetK* and *mef(A)* genes were detected in several *Streptococcus* species (see Supplementary Table S1). In addition, the presence of *tetM* gene was presumptively found in 81.8% of *Streptococcus* spp. and 8 out of 11 of them carried the *pmrA* gene.

In this study, the high rate of MDR was most predominant in the patient’s tracheal samples with 46.9% among the MDR strains. In contrast, many non-MDR strains were originated from blood specimens with 42.3%.

According to the antimicrobial susceptibility testing (AST) data, 49 out of 127 isolates demonstrated phenotypically resistant traits to at least three antibiotic classes, which are in accordance with the multidrug-resistant criteria (Supplementary Table S1). Based on the data, it was also noted that a total of 19 *S. aureus* isolates were identified as MSSA, and 11 isolates were recognized as MRSA (as shown in Table 4). As expected, all MRSA strains were resistant to benzylpenicillin and oxacillin. Both MSSA and MRSA strains were susceptible to quinupristin/dalfopristin, linezolid, vancomycin, tigecycline and nitrofurantoin. Among the *S. aureus* isolates, MRSA had higher rates of resistance towards antibiotics compared to MSSA. For other Gram-positive bacteria, such as *Streptococcus* species, there was no significant susceptibility data available as routine screening for antimicrobial susceptibility was unnecessary for them. Only 3 of the 11 *Streptococcus* isolates were subjected to AST, which showed resistance to clindamycin and tetracycline.

For Gram-negative bacteria, the phenotypic resistance analysis revealed that 46 (100%) of the *K. pneumoniae* isolates were susceptible to amikacin and 2 (4.3%) were shown to have resistance phenotype to carbapenem (such as imipenem and meropenem). Some of the *K. pneumoniae* isolates (32.6%) were susceptible for cefazolin, and it was noted that 37% of them had susceptibility towards levofloxacin. In contrast, more than half of the total number of isolates of *A. baumannii* and *P. aeruginosa*, demonstrated susceptibility to levofloxacin. *P. aeruginosa* isolates showed high percentages of susceptibility (>70%) to the majority of the tested antibiotics, including imipenem, meropenem, ceftazidime, cefepime and levofloxacin as shown in Table 4. In this present study, the rate of non-susceptibility among *A. baumannii* isolates was as follows: 100% (*n* = 7) for cefazolin, 42.9% (*n* = 3) for tobramycin, ampicillin/sulbacyam, piperacillin/tazobactam, imipenem, meropenem, ceftazidime, cefepime, ciprofloxacin and levofloxacin, and 28.6% (*n* = 2) for trimethoprim/sulfamethoxazole. Some of the *A. baumannii* isolates sampled from the tracheal aspirates showed a similar antimicrobial susceptibility pattern to those from the bronchial aspirate, blood specimens and wounds (Table S1). However, two of *A. baumannii* isolates from bronchial aspirate and tracheal aspirate were resistant to all tested antibiotics by the IJN. It was also observed that ceftriaxone and levofloxacin only possessed intermediate effectiveness against *A. baumannii* isolates.

### 3.3. Ability of Biofilm Formation

In biofilm assays, 44 out of 127 isolates were categorized as biofilm producers. Among them, 11.3% were strong biofilm producers. Furthermore, 12 of the 44 biofilm producers showed moderate biofilm production and the remaining 27(61.4%) demonstrated weak biofilm production. Importantly, 31.8% of biofilm producers carried multiple antibiotic resistance genes. The percentage ability in forming biofilms among the MDR isolates was as follows: 57.1% for weak biofilm producers; 28.8% for moderate biofilm producers and 14.3% for strong biofilm producers. Interestingly, there were two strong biofilm producers identified as multidrug-resistant strains according to their phenotypic resistance profiles. Both were resistant to cefazolin, ceftazidime and cefepime. Further details of each biofilm producer are summarised in Supplementary Table S1.

## 4. Discussion

The rising incidence of microbial resistance to antibiotics has caused considerable challenges in the management of postoperative infections due to the lack of effective therapeutic options. This rise in incidence has been leading to increased morbidity and mortality rates in postoperative infections, prolonged hospital stays and consequently higher management costs. The majority of the causative agents associated with postoperative infections are the ESCAPE (*Enterococcus faecium, Staphylococcus aureus, Clostridium difficille, Acinetobacter baumannii, Pseudomonas aeruginosa*, and Enterobacteriaceae species) group of pathogens [54]. These organisms are capable to ‘escape’ antibacterial actions of the current range of antibiotics. In addition, some of them are normal flora to humans and may acquire antibiotic resistance elements via inter- or intraspecies gene transfer between them. Meanwhile, misuse of antibiotics has also caused enormous damage in aggravating antimicrobial resistance, which now seems to develop faster than usual.

Unlike previous reports [55,56,57], which stated that the majority of postoperative infections are caused by *S. aureus*, our study showed that most of the cardiac surgery patients were prone to severe postoperative infections caused by *K. pneumoniae*. This observation was consistent with the findings from Hope et al. [58]. Besides, another study also reported that *K. pneumoniae* was one of the leading isolates in postoperative infections, which accounted for 50% cases, followed by *S. aureus* (27.8%) [59]. In the present study, we found that *K. pneumoniae* was the most predominant organism, which caused postoperative infections in 36.2% of subjects, followed by *P. aeruginosa* (26%), paralleling with the study-findings reported by Ali et al. [60]. Ali et al. stated that superficial incisional surgical site infections, which were the most common type of postoperative infections in surgical patients, were predominantly caused by *K. pneumoniae* [60]. The virulence factor of *K. pneumoniae* might be the possible explanation for the high number of *K. pneumoniae* infection contributors in our study. The outer layer of *K. pneumoniae* inhibits phagocytosis by reducing the amount of complement component 3 (C3) that binds to the surface of *K. pneumoniae*, as demonstrated by Guadalupe et al. [61]. Therefore, most of the polymorphonuclear cells lack the killing efficacy when *K. pneumoniae* was not opsonised by C3. Similarly, *P. aeruginosa* was also shown to be able to evade the host immunity (especially in those who are immunosuppressed and are hospitalized) via its slime production, which prevents the complement components deposition that commonly leads to phagocytosis [62,63,64]. Therefore, *P. aeruginosa* was identified as the second most dominant causative bacteria isolated from our study, which was followed by *S. aureus*. It is not surprising that *S. aureus* is one of the isolates harvested from these patients because it is well-known as an opportunistic bacterium associated with bacteraemia and surgical site infection. In contrast, only 11 isolates of *Streptococcus* spp. were collected from cases of postoperative infection. All these streptococcal isolates were isolated from blood specimens, where 81.8% of them were beta-haemolytic streptococcus. The low number of *Streptococcus* spp. in our total specimens is consistent with the study conducted by Chaudhary et al. [55] in a surgical ward of Bharatour Hospital, Chitwan, Nepal, which reported only 1.5% of *Streptococcus* spp. as observed under surgical site infections.

In the present study, the antibiotic susceptibility data were compared with the antibiotic resistance gene patterns in *S. aureus, K. pneumoniae, A. baumanni, P. aeruginosa* and *Streptococcus* spp. isolates. To our knowledge, this is the first report of bacterial genotypic and phenotypic antibiotic resistance patterns from our local heart specialist institution IJN. The antibiotic susceptibility results showed a high resistance rate among the *K. pneumoniae* isolates to ampicillin/sulbactam (69.6%), which is the highest among all the conventional antibiotics. All *K. pneumoniae* isolates were found to be positive to *bla*_CTX-M_ and bla*_SHV_* as confirmed by PCR studies, unlike the report of Kiratisin et al., which stated that the majority of *K. pneumoniae* (99.2%) carried the *bla*_CTX-M_ gene while 87.4% of ESBL-producing *K. pneumoniae* carried the *bla*_SHV_ gene [65]. The combined presence of three ESBL genes (*bla*_CTX-M_, *bla*_GES_ and *bla*_SHV_) in the same *K. pneumoniae* isolates were 80.4%. In contrast, a low percentage of multiple ESBL genes in ESBL-producing *K. pneumoniae* was reported in the findings from Mohsen et al. (28%) and Apisarnthanarak et al. (24%) [66,67]. Although all the *K. pneumoniae* isolates harboured the *bla*_SHV_ gene that was responsible for cephalosporin resistance, some of the *K. pneumoniae* isolates were still susceptible to cefepime (41.3%), ceftriaxone (37.0%), cefazolin (32.6%) and ceftazidime (34.8%). There was another study, which demonstrated that, the absence of cephalosporin phenotypic properties in the *bla*_SHV_ gene-carrying *K. pneumoniae* isolates could be caused by the growth conditions and environment of the bacteria. Changes in the growth conditions may affect the bacteria’s physiological adaptations, resulting in changes in its metabolic process [68]. The regulatory proteins of bacteria are always controlled by nutrient availability and its metabolites. In *K. pneumoniae,* some of the amino acids were found to bind to the repressors, which in turn had bound to the regulatory sites on DNA, which resulted in *bla*_SHV_ gene suppression.

As expected, a total of 11 (36.7%) *S. aureus* isolates were found to be methicillin-resistant strains (MRSA), where all of them were resistant to benzylpenicillin and oxacillin. Interestingly, 9 of the MSSA strains, which were susceptible to oxacillin also contained the gene, which confers resistance to oxacillin, called *mec*A. This means some staphylococcal isolates could still be killed by oxacillin, despite the presence of *mec*A. Similarly, Proulx et al. revealed a similar finding where they concluded that this phenomenon may be due to genetically inactivated *mec*A in MSSA. This phenomenon is called silencing of antibiotic resistance by mutation (SARM). SARM results in non-functional antibiotic resistance protein as mutation is found at its genetic coding region, which affects its transcription or translation functionalities [57]. Other than the *mec*A gene, some of the MSSA isolates, which expressed its erythromycin susceptible phenotype, were found to harbour the *ermA* and *ermC* genes. Supposedly, the *erm* genes encode the erythromycin-resistance methyltransferase to initiate the ribosomal RNA methylation. The methylation of ribosomal RNA will inhibit erythromycin-binding resulting in MSSA resistance to erythromycin [69]. Similar observations were reported by Zmantar et al. [70] and Sekiguchi et al. where they had reported the absence of the resistance phenotype towards erythromycin in isolates, in spite of the presence of *erm* genes [71] Sekiguchi et al. suggested that there might be a mutational event in the coding or promoter region of the *erm gene*, which was carried by *S. aureus* strains as evidenced via PCR analysis [71]. In addition to the *ermA* or *ermC* gene, the presence of the *erm*B gene was also detected in this study for *S. aureus;* but only 3.3%, which corroborates with the findings of Eady et al. in clinical *S. aureus* isolates [72].

Infection with multidrug-resistant bacteria has been associated with significant morbidity and mortality. In this study, we found that the prevalence of multidrug-resistant (MDR) strains was 38.6% (49 out of 127 isolates), which is a significant proportion that can affect the treatment of patients with postoperative infections. Cefazolin showed the highest resistance rate (81.6%) among the multidrug-resistant isolates. Another study conducted at a national hospital in an East African country depicted a much higher rate of MDR (63%) among the pathogens that caused postoperative infections [73]. Among MDR *P. aeruginosa,* piperacillin exhibited the highest resistance pattern (100%). Furthermore, Tavajjohi et al. also reported that 89.3% of MDR *P. aeruginosa* isolates in their study were resistant to piperacillin. [74] Multidrug-resistant status is one of the significant determinants of hospital mortality. Hospital-acquired infections associated with *P. aeruginosa* and *A. baumannii* were 8.4% in the study conducted by Motbainor et al. [75]. All the isolates of MDR *P. aeruginosa* and *A. baumannii* were resistant to cefazolin and six of the nine MDR *P. aeruginosa* isolates were resistant to piperacillin/tazobactam. The cefazolin resistance rate was found to be lower compared to the report from Northwest Ethiopia [75]. Most recently, a study has shown that the presence of the *bla*_KPC-2_ gene in *P. aeruginosa* could be associated with its susceptibility to ceftazidime-avibactam by increasing the minimum inhibitory concentration value [76]. In the present study, 27.3% of *P. aeruginosa* were revealed to be resistant to ceftazidime. The high number of MDR among *P. aeruginosa* and *A. baumannii* observed in postoperative patients in IJN was probably related to cross-contamination of these bacteria in the hospital environment or some other unknown factors, which is not the focus of this present study.

From the 127 bacterial isolates screened for biofilm formation, 44 of them were detected to be biofilm producers. This feature may contribute to the development of antibiotic resistance and plays an important role in evading the effect of immune defense mechanism. *K. pneumoniae* 13 (28.2%), *P. aeruginosa* 11(33.3%), *S. aureus* 9 (30%), *Streptococcus* spp. 6 (54.5%), and *A. baumannii* 5 (71.4%) were among the leading biofilm producers as found in this study. However, the number of biofilm producers that were found in this study was not as high as in the study reported by Barsoumian et al., which reported 60.4% of biofilm producers detected in clinical samples [77]. Biofilms could be an important concern since there are studies that showed that biofilms play a key role in bacterial transmissions in different areas of a hospital. These may also include the cross-transmission of bacteria within a clinical setting or high-contact equipment items such as stethoscopes, ventilators, and phones. Healthcare workers, especially with poor hand hygiene, may be responsible for further environmental contamination as they work across different patient zones every day [78,79]. Besides, horizontal gene transfer, which is the process for antibiotic resistance gene interchange, frequently happens in biofilms [80]. This causes genetic variation in bacteria, consequently resulting in superbug infection-bacteria development. Several studies have revealed that the rate of horizontal gene transfer of antibiotic-resistant genes is typically higher in the biofilm community compared to those in planktonic cultures [81,82]. Hence, biofilm is believed to be the main antibiotic-resistant gene reservoir for genetic exchange among bacterial species. Nevertheless, the biofilm data in our study is non-conclusive to confirm if the detected MDR strains as aforementioned were a consequence of the presence of these biofilm producers.

## 5. Conclusions

Taken together, the findings of this present study could further guide and advance the knowledge base of existing antibiotic resistance principles and surgical procedure guidelines, which may, in turn, improve the quality of postoperative care for patients. Furthermore, our study also reports the high prevalence of postoperative infection cases and the emergence of MDR bacteria, which have highlighted the challenges and difficulties in the management of postoperative infections. Although the number of MDR isolates and biofilm producer rates were relatively low in this present study, prudent antimicrobial use and effective infection prevention and control (IPC) strategies are important to prevent further development of multidrug resistance and the emergence of newer biofilm-producing strains.

## Figures and Tables

**Table 1:** PCR primers and programme specifications for the targeted genes in the respective bacteria [38,39,40,41,42,43,44,45,46,47,48,49,50,51,52,53].

Isolates	Genes	Primers (5’−3’)	PCR Products (Base Pair)	PCR Programme Specifications				
				Initial Denaturation	Denaturation	Annealing	Extension	Final Extension
*S. aureus* *Streptococcus* spp.	*ermA*	AAG CGG TAA ACC CCT CTG A; TTC GCA AAT CCC TTC TCA AC	190 bp	94 °C, 3 min	35 cycles of 94 °C, 30 s	55 °C, 45 s	72 °C, 45 s	72 °C, 10 min
*S. aureus* *Streptococcus* spp.	*ermB*	CTATCTGATTGTTGAAGAAGGATT; GTTTACTCTTGGTTTAGGATGAAA	142 bp	94 °C, 3 min	35 cycles of 94 °C, 30 s	55 °C, 45 s	72 °C, 45 s	72 °C, 10 min
*S. aureus* *Streptococcus* spp.	*ermC*	AAT CGT CAA TTC CTG CAT GT; TAA TCG TGG AAT ACG GGT TTG	299 bp	94 °C, 3 min	35 cycles of 94 °C, 30 s	55 °C, 45 s	72 °C, 45 s	72 °C, 10 min
*S. aureus* *Streptococcus* spp.	*tetK*	GTA GCG ACA ATA GGT AAT AGT; GTA GTG ACA ATA AAC CTC CTA	360 bp	94 °C, 3 min	35 cycles of 94 °C, 30 s	55 °C, 45 s	72 °C, 45 s	72 °C, 10 min
*S. aureus* *Streptococcus* spp.	*tetM*	AGT GGA GCG ATT ACA GAA; CAT ATG TCC TGG CGT GTC TA	158 bp	94 °C, 3 min	35 cycles of 94 °C, 30 s	55 °C, 45 s	72 °C, 45 s	72 °C, 10 min
*S. aureus* *Streptococcus* spp.	*msrA*	GAA GCA CTT GAG CGT TCT; CCT TGT ATC GTG TGA TGT	287 bp	95 °C, 2 min	30 cycles of 94 °C, 30 s	50 °C, 30 s	72 °C, 30 s	72 °C, 4 min
*S. aureus* *Streptococcus* spp. *K. pneumoniae* *A. baumannii* *P. aeruginosa*	*mcr-1*	CGGTCAGTCCGTTTGTTC; CTTGGTCGGTCTGTAGGG	309 bp	94 °C, 15 min	25 cycles of 94 °C, 30 s	58 °C, 90 s	72 °C, 1 min	72 °C, 10 min
*S. aureus*	*aacA-aphD*	TAA TCC AAG AGC AAT AAG GGC; GCC ACA CTA TCA TAA CCA CTA	227 bp	94 °C, 3 min	35 cycles of 94 °C, 30 s	55 °C, 45 s	72 °C, 45 s	7 2 °C, 10 min
*S. aureus*	*mecA*	AAAATCGATGGTAAAGGTTGGC; AGTTCTGCAGTACCGGAT TTGC	533 bp	95 °C, 1 min	35 cycles of 95 °C, 1 min	54 °C, 1 min	72 °C, 1 min	72 °C, 5 min
*S. aureus*	*vanA*	GGGAAAACGACAATTGC; GTACAATGCGGCCGTTA	732 bp	94 °C, 2 min	30 cycles of 94 °C, 1 min	54 °C, 1 min	72 °C, 1 min	72 °C, 10 min
*S. aureus*	*vanB*	ATGGGAAGCCGATAGTC; GATTTCGTTCCTCGACC	635 bp	94 °C, 2 min	30 cycles of 94 °C, 1 min	54 °C, 1 min	72 °C, 1 min	72 °C, 10 min
*S. aureus* *Streptococcus* spp.	*mef(A)*	AGT ATC ATT AAT CAC TAG TGC; TTC TTC TGG TAC AAA AGT GG	348 bp	95 °C, 5 min	35 cycles of 95 °C, 30 s	54 °C, 30 s	72 °C, 1 min	72 °C, 5 min
*S. aureus* *Streptococcus* spp.	*mef(E)*	AGT ATC ATT AAT CAC TAG TGC; TTC TTC TGG TAC AAA AGT GG	1218 bp	94 °C, 5 min	35 cycles of 94 °C, 30 s	50 °C, 30 s	72 °C, 90 s	72 °C, 5 min
*A. baumannii* *Streptococcus* spp.	*pmrA*	TCCAGTATGGGCTTTTCCAG; CCAATCCAAAGAGGAAACGA	178 bp	95 °C, 9 min	40 cycles of 95 °C, 15 s	53.7 °C, 1 min	72 °C, 1 min	72 °C, 5 min
*Streptococcus* spp.	*catQ*	TAGAAAGCCATACTTTGAGC; CATGATGCACCTGTACAGAC	536 bp	95 °C, 2 min	35 cycles of 95 °C, 30 s	50 °C, 30 s	72 °C, 30 s	72 °C, 5 min
*Streptococcus* spp.	*InuB*	CCTACCTATTGTTTGTGGAA; ATAACGTTACTCTCCTATTC	944 bp	94 °C, 5 min	35 cycles of 94 °C, 45 s	54 °C, 45 s	72 °C, 1 min	72 °C, 5 min
*K. pneumoniae* *A. baumannii*	*bla*_CTX-M_	TTTGCGATGTGCAGTACCAGTAA; CGATATCGTTGGTGGTGCCATA	544 bp	95 °C, 5 min	35 cycles of 95 °C, 30 s	51 °C, 45 s	72 °C, 1 min	72 °C, 10 min
*K. pneumoniae* *A. baumannii*	*bla*_SHV_	ATGCGTTATATTCGCCTGTG; TGCTTTGTTATTCGGGCCAA	753 bp	95 °C, 5 min	35 cycles of 95 °C, 30 s	60 °C, 45 s	72 °C, 1 min	72 °C, 10 min
*K. pneumoniae* *A. baumannii*	*bla*_KPC-2_	GTATCGCCGTCTAGTTCTG; CCTTGAATGAGCTGCACAGTGG	209 bp	95 °C, 3 min	40 cycles of 95 °C, 5 s	50 °C, 30 s	72 °C, 1 min	72 °C, 5 min
*K. pneumoniae* *A. baumannii*	*bla*_GES_	GTTTTGCAATGTGCTCAACG; TGCCATAGCAATAGGCGTAG	371 bp	95 °C, 5 min	35 cycles of 95 °C, 30 s	53 °C, 45 s	72 °C, 1 min	72 °C, 10 min
*K. pneumoniae* *A. baumannii*	*bla*_IMP_	GTTTATGTTCATACATCG; GGTTTAACAAAACAACCAC	440 bp	95 °C, 5 min	35 cycles of 95 °C, 30 s	45 °C, 45 s	72 °C, 1 min	72 °C, 10 min
*K. pneumoniae* *A. baumannii*	*bla*_VIM_	TTTGGTCGCATATCGCAACG; CCATTCAGCCAGATCGGCAT	500 bp	95 °C, 5 min	35 cycles of 95 °C, 30 s	66 °C, 45 s	72 °C, 1 min	72 °C, 10 min
*K. pneumoniae* *A. baumannii*	*bla*_SPM-1_	CCTACAATCTAACGGCGACC; TCGCCGTGTCCAGGTATAAC	674 bp	95 °C, 5 min	30 cycles of 95 °C, 1 min	40 °C, 1 min	68 °C, 1 min	68 °C,5 min
*K. pneumoniae* *A. baumannii*	*bla*_NDM-1_	GGGCAGTCGCTTCCAACGGT; GTAGTGCTCAGTGTCGGCAT	475 bp	94 °C, 3 min	40 cycles of 94v, 30 s	60 °C, 30 s	72 °C, 30 s	72 °C, 3 min
*K. pneumoniae* *A. baumannii*	*bla*_OXA-23_	CCCCGAGTCAGATTGTTCAAGG; TAC GTCGCGCAAGTTCCTGA	330 bp	95 °C, 15 min	30 cycles of 94 °C, 30 s	57 °C, 90 s	72 °C, 90 s	72 °C, 10 min
*K. pneumoniae* *A. baumannii*	*bla*_OXA-24_	CACCTATGGTAATGCTCTTGC; CAACCTACCTGTGGAGTAACAC	501 bp	95 °C, 3 min	40 cycles of 95 °C, 5 s	50 °C, 30 s	72 °C, 1 min	72 °C, 5 min
*K. pneumoniae* *A. baumannii*	*bla*_OXA-58_	GGGGCTTGTGCTGAGCATAGT; CCACTTGCCCATCTGCCTTT	688 bp	95 °C, 15 min	30 cycles of 94 °C, 30 s	57 °C, 90 s	72 °C, 90 s	72 °C, 10 min

**Table 2:** Distribution of isolated bacteria across various infected body sites.

	Blood	Pus/Wound	Tracheal	Tissue	Bronchial	Urine	Sputum
*S. aureus*	18	9	2	1	0	0	0
*K. pneumoniae*	35	5	4	0	1	0	1
*A. baumannii*	5	0	1	0	0	1	0
*P. aeruginosa*	2	8	20	2	1	0	0
*Streptococcus* spp.	11	0	0	0	0	0	0

**Table 3:** Antibiotic resistance genes detected in clinical isolates.

	*S. Aureus*	*K. Pneumoniae*	*A. Baumannii*	*P. Aeruginosa*	*Streptococcus* spp.
	(*n* = 30)	(*n* = 46)	(*n* = 7)	(*n* = 33)	(*n* = 11)
	Number (%)	Number (%)	Number (%)	Number (%)	Number (%)
*ermA*	12 (40)	-	-	-	0 (0)
*ermB*	1 (3.3)	-	-	-	5 (45.5)
*ermC*	26 (86.7)	-	-	-	0 (0)
*tetK*	0 (0)	-	-	-	1 (9.1)
*tetM*	21 (70)	-	-	-	9 (81.8)
*msrA*	0 (0)	-	-	-	0 (0)
*mcr-1*	0 (0)	1 (2.17)	0 (0)	0 (0)	0 (0)
*aacA-aphD*	19 (63.3)	-	-	-	-
*mecA*	20 (66.7)	-	-	-	-
*vanA*	0 (0)	-	-	-	-
*vanB*	0 (0)	-	-	-	-
*mef(A)*	0 (0)	-	-	-	1 (9.1)
*mef(E)*	0 (0)	-	-	-	0 (0)
*pmrA*	-	-	2 (28.6)	-	8 (72.7)
*catQ*	-	-	-	-	0 (0)
*InuB*	-	-	-	-	0 (0)
*bla*_CTX-M_	-	46 (100)	3 (42.9)	33 (100)	-
*bla*_SHV_	-	46 (100)	6 (85.7)	31 (93.9)	-
*bla*_KPC-2_	-	0 (0)	1 (16.7)	7 (21.2)	-
*bla_GES_*	-	37 (80.4)	2 (28.6)	33 (100)	-
*bla*_IMP_	-	0 (0)	1 (16.7)	0 (0)	-
*bla*_VIM_	-	7 (15.2)	1 (16.7)	0 (0)	-
*bla*_SPM-1_	-	0 (0)	0 (0)	2 (6.1)	-
*bla*_NDM-1_	-	9 (19.6)	3 (42.9)	1 (3.0)	-
*bla*_OXA-23_	-	5 (10.9)	5 (71.4)	220 (66.7)	-
*bla*_OXA-24_	-	0 (0)	0 (0)	0 (0)	-
*bla*_OXA-58_	-	3 (6.5)	0 (0)	0 (0)	-

**Table 4:** Susceptible profiles of isolates to β-lactam and non-β-lactam antibiotics.

Drug	*S. aureus*	*K. pneumoniae*	*A. baumanni*	*P. aeruginosa*	*Streptococcus* spp.
	(*n* = 30)	(*n* = 46)	(*n* = 7)	(*n* = 33)	(*n* = 11)
	Number (%)	Number (%)	Number (%)	Number (%)	Number (%)
Amikacin	-	46 (100)	-	32 (97)	-
Tobramycin	-	26 (56.5)	4 (57.1)	32 (97)	-
Gentamicin	29 (96.7)	-	-	-	-
Benzylpenicillin	5 (16.7)	-	-	-	3 (27.3)
Oxacillin	19 (63.3)	-	-	-	-
Ampicillin	-	-	-	-	3 (27.3)
Ampicillin/sulbactam	-	14 (30.4)	4 (57.1)	-	-
Piperacillin/tazobactam	-	25 (54.3)	4 (57.1)	22 (66.7)	-
Ertapenem	-	43 (93.5)	-	-	-
Imipenem	-	44 (95.7)	4 (57.1)	24 (72.7)	-
Meropenem	-	44 (95.7)	4 (57.1)	24 (72.7)	-
Cefotetan	-	-	-	-	1 (9.1)
Cefazolin	-	15 (32.6)	0 (0)	0 (0)	-
Ceftazidime	-	16 (34.8)	4 (57.1)	24 (72.7)	-
Ceftriaxone	-	17 (37.0)	-	-	1 (9.1)
Cefepime	-	19 (41.3)	4 (57.1)	24 (72.7)	-
Chloramphenicol	-	-	-	-	1 (9.1)
Levofloxacin	19 (63.3)	17(37.0)	4 (57.1)	24 (72.7)	3 (27.3)
Teicoplanin	-	-	-	-	1 (9.1)
Vancomycin	30 (100)	-	-	-	3 (27.3)
Tigecycline	30 (100)	-	-	-	3 (27.3)
Clindamycin	21 (70)	-	-	-	2 (18.2)
Erythromycin	20 (66.7)	-	-	-	1 (9.1)
Nitrofurantoin	30 (100)	9 (19.6)	-	-	2 (18.2)
Linezolid	30 (100)	-	-	-	3 (27.3)
Ciprofloxacin	19 (63.3)	17 (37.0)	4 (57.1)	26 (78.8)	-
Moxifloxacin	19 (63.3)	-	-	-	3 (27.3)
Rifampicin	29 (96.7)	-	-	-	-
Quinupristin/Dalfoprostin	30 (100)	-	-	-	2 (6.1)
Trimethoprim/Sulfamethoxazole	29 (96.7)	19 (41.3)	5 (71.4)	-	-
Tetracycline	26 (86)	-	-	-	2 (18.2)

## Data Availability

The data presented in this study are available in Supplementary Table S1.

## Supplementary Materials

The following are available online at https://www.mdpi.com/article/10.3390/ijerph18189828/s1, Table S1: Degree of biofilm formation and resistance gene among the patient’s specimen with the resistance pattern of bacterial isolates to 11 antibiotic classes.
