# Supplementary material for: Characterisation of Bacterial Isolates from Infected Post-Operative Patients in a Malaysian Tertiary Heart Care Centre
Source: Int J Environ Res Public Health. 2021 Sep 17;18(18):9828. doi: 10.3390/ijerph18189828 (PMC8471342; doi:10.3390/ijerph18189828)
Supplement: Supplementary file 1 [file ijerph-18-09828-s001.zip › ijerph-1364367-supplementary.pdf]

TableS1: Degree of biofilm formation and resistance gene among the patient's specimen with the resistance pattern of bacterial isolates to 11 antibiotic classes.

Isolates	Source	Antibiotic-resistant phenotypes	Antibiotic-resistant genotypes
Staphylococcus aureus			
SA1	Tracheal aspirate		*ermA, ermC, tetM, aacA-aphD*
SA2	Pus/wound		*ermA, ermC, tetM, aacA-aphD*
SA3	Blood		*ermA, ermC, tetM, aacA-aphD*
SA4	Blood		*ermA, ermC, tetM, aacA-aphD*
SA5	Blood		*ermA, ermC, tetM, mecA*
SA6	Blood		*ermA, ermC, mecA*
SA7	Pus/wound		*ermA, aacA-aphD, mecA*
SA8	Blood		*ermA, tetM*
SA9[++]	Wound		*ermC, tetM, mecA*
SA10	Pus/wound		*ermB, ermC, tetM, aacA-aphD, mecA*
SA11	Blood		*ermA, ermC, tetM, aacA-aphD*
SA12	Blood		*ermA, ermC, tetM, mecA*
SA13	Pus/wound		*ermA, ermC, tetM, aacA-aphD, mecA*
SA14	Blood		*ermC, tetM, mecA*
SA15[+]	Blood		*ermC*
SA16	Blood		*ermC, mecA*
SA17	Pus/wound		*mecA*
SA18[+]	Tracheal aspirate		*ermC, aacA-aphD, mecA*
SA19[+]	Pus/wound		*ermC, tetM*
SA20[+]	Pus/wound		*ermA, ermC, tetM, aacA-aphD, mecA*
SA21[+++]	Blood		*ermC, tetM, aacA-aphD, mecA*
SA22	Blood		*ermC, aacA-aphD, mecA*

SA23	Pus/wound		*ermC, tetM, aacA-aphD, mecA*
SA24[+]	Blood		*ermC, tetM, aacA-aphD, mecA*
SA25	Blood		*ermC, tetM, aacA-aphD, mecA*
SA26	Blood		*ermC, aacA-aphD, mecA*
SA27	Tissue		*ermC, tetM, aacA-aphD, mecA*
SA28	Blood		*ermC, tetM, aacA-aphD, mecA*
SA29[+]	Blood		*ermC, tetM*
SA30[+]	Blood		*tetM, aacA-aphD*
		Klebsiella pneumonia	
KP1	Tracheal aspirate		*bla*$_{\text{CTX-M}}$, *bla*$_{\text{SHV}}$, *bla*$_{\text{GES}}$,
KP2	Tracheal aspirate		*bla*$_{\text{CTX-M}}$, *bla*$_{\text{SHV}}$, *bla*$_{\text{GES}}$,
KP3	Pus/wound		*bla*$_{\text{CTX-M}}$, *bla*$_{\text{SHV}}$, *bla*$_{\text{GES}}$,
KP4	Pus/wound		*bla*$_{\text{CTX-M}}$, *bla*$_{\text{SHV}}$, *bla*$_{\text{GES}}$, *bla*$_{\text{VIM}}$
KP5	Tracheal aspirate		*bla*$_{\text{CTX-M}}$, *bla*$_{\text{SHV}}$, *bla*$_{\text{GES}}$
KP6	Tracheal aspirate		*bla*$_{\text{CTX-M}}$, *bla*$_{\text{SHV}}$, *bla*$_{\text{GES}}$, *bla*$_{\text{VIM}}$
KP7[+]	Urine		*bla*$_{\text{CTX-M}}$, *bla*$_{\text{SHV}}$
KP8	Urine		*bla*$_{\text{CTX-M}}$, *bla*$_{\text{SHV}}$
KP9	Tissue/biopsy		*bla*$_{\text{CTX-M}}$, *bla*$_{\text{SHV}}$, *bla*$_{\text{GES}}$
KP10	Tracheal aspirate		*bla*$_{\text{CTX-M}}$, *bla*$_{\text{SHV}}$, *bla*$_{\text{GES}}$,
KP11	Urine		*bla*$_{\text{CTX-M}}$, *bla*$_{\text{SHV}}$, *bla*$_{\text{GES}}$
KP12[++]	Tracheal aspirate		*bla*$_{\text{CTX-M}}$, *bla*$_{\text{SHV}}$, *bla*$_{\text{GES}}$
KP13	Tracheal aspirate		*bla*$_{\text{CTX-M}}$, *bla*$_{\text{SHV}}$, *bla*$_{\text{GES}}$, *bla*$_{\text{OXA-58}}$
KP14	Tracheal aspirate		*bla*$_{\text{CTX-M}}$, *bla*$_{\text{SHV}}$, *bla*$_{\text{GES}}$
KP15	Tracheal aspirate		*bla*$_{\text{CTX-M}}$, *bla*$_{\text{SHV}}$, *bla*$_{\text{GES}}$, *bla*$_{\text{OXA-58}}$
KP16	Blood		*bla*$_{\text{CTX-M}}$, *bla*$_{\text{SHV}}$, *bla*$_{\text{GES}}$
KP17	Pus/wound		*bla*$_{\text{CTX-M}}$, *bla*$_{\text{SHV}}$, *bla*$_{\text{GES}}$
KP18[++]	Blood		*bla*$_{\text{CTX-M}}$, *bla*$_{\text{SHV}}$, *bla*$_{\text{GES}}$,
KP19	Tissue/biopsy		*bla*$_{\text{CTX-M}}$, *bla*$_{\text{SHV}}$, *bla*$_{\text{GES}}$
KP20	Urine		*bla*$_{\text{CTX-M}}$, *bla*$_{\text{SHV}}$

KP21^{++}	Tracheal aspirate		$bla_{\text{CTX-M}}$, bla_{SHV}, bla_{GES}, bla_{VIM}
KP22	Tracheal aspirate		$bla_{\text{CTX-M}}$, bla_{SHV}, bla_{GES}
KP23^{+}	Blood		$bla_{\text{CTX-M}}$, bla_{SHV}, bla_{GES}, bla_{VIM}
KP24	Pus/wound		*mcr-1*, $bla_{\text{CTX-M}}$, bla_{SHV}, bla_{GES}
KP25	Tracheal aspirate		$bla_{\text{CTX-M}}$, bla_{SHV}, bla_{GES}, bla_{VIM}, $bla_{\text{NDM-1}}$, $bla_{\text{OXA-23}}$
KP26	Tracheal aspirate		$bla_{\text{CTX-M}}$, bla_{SHV},
KP27	Pus/wound		$bla_{\text{CTX-M}}$, bla_{SHV}, bla_{GES}, $bla_{\text{OXA-23}}$
KP28	Tracheal aspirate		$bla_{\text{CTX-M}}$, bla_{SHV}, bla_{GES}, $bla_{\text{NDM-1}}$, $bla_{\text{OXA-23}}$
KP29	Urine		$bla_{\text{CTX-M}}$, bla_{SHV}, bla_{GES}
KP30	Urine		$bla_{\text{CTX-M}}$, bla_{SHV}, bla_{GES},
KP31^{+}	Tissue/biopsy		$bla_{\text{CTX-M}}$, bla_{SHV}, bla_{GES},
KP32^{++}	Tracheal aspirate		$bla_{\text{CTX-M}}$, bla_{SHV}, bla_{GES}, $bla_{\text{NDM-1}}$
KP33	Bronchial aspirate		$bla_{\text{CTX-M}}$, bla_{SHV}, $bla_{\text{NDM-1}}$
KP34	Tracheal aspirate		$bla_{\text{CTX-M}}$, bla_{SHV}, $bla_{\text{NDM-1}}$
KP35^{+}	Tracheal aspirate		$bla_{\text{CTX-M}}$, bla_{SHV}, bla_{GES}, $bla_{\text{NDM-1}}$
KP36	Tracheal aspirate		$bla_{\text{CTX-M}}$, bla_{SHV}, bla_{GES}, bla_{VIM}
KP37	Blood		$bla_{\text{CTX-M}}$, bla_{SHV}, bla_{GES}
KP38^{+}	Tracheal aspirate		$bla_{\text{CTX-M}}$, bla_{SH}
KP39^{++}	Tracheal aspirate		$bla_{\text{CTX-M}}$, bla_{SHV}, bla_{GES},
KP40^{+++}	Sputum		$bla_{\text{CTX-M}}$, bla_{SHV}, bla_{GES},
KP41	Tracheal aspirate		$bla_{\text{CTX-M}}$, bla_{SHV}, bla_{GES},
KP42^{+}	Blood		$bla_{\text{CTX-M}}$, bla_{SHV}, bla_{VIM}, $bla_{\text{NDM-1}}$, $bla_{\text{OXA-23}}$
KP43^{+++}	Tracheal aspirate		$bla_{\text{CTX-M}}$, bla_{SHV}
KP44	Urine		$bla_{\text{CTX-M}}$, bla_{SHV}, bla_{GES}, $bla_{\text{NDM-1}}$, $bla_{\text{OXA-23}}$, $bla_{\text{OXA-58}}$
KP45	Blood		$bla_{\text{CTX-M}}$, bla_{SHV}, $bla_{\text{NDM-1}}$
KP46	Tracheal aspirate		$bla_{\text{CTX-M}}$, bla_{SHV}, bla_{GES},
	Acinetobacter baumannii		
AB1	Tracheal aspirate		*pmrA*, $bla_{\text{CTX-M}}$, bla_{GES}, bla_{VIM}, $bla_{\text{NDM-1}}$, $bla_{\text{OXA-23}}$

AB2	Tracheal aspirate		bla_{SHV}, bla_{IMP},
AB3^{++}	Tracheal aspirate		$bla_{CTX\text{-}M}$, bla_{SHV}, $bla_{OXA\text{-}23}$
AB4^{+}	Tracheal aspirate		bla_{SHV}, $bla_{NDM\text{-}1}$, $bla_{OXA\text{-}23}$
AB5^{+}	Bronchial aspirate		bla_{SHV}, bla_{GES}, $bla_{OXA\text{-}23}$
AB6^{++}	Blood		bla_{SHV}, $bla_{KPC\text{-}2}$
AB7^{+}	Pus/wound		*pmrA*, $bla_{CTX\text{-}M}$, bla_{SHV} , bla_{GES}, $bla_{NDM\text{-}1}$, $bla_{OXA\text{-}23}$
Pseudomonas aeruginosa			
PA1	Tracheal aspirate		$bla_{CTX\text{-}M}$, bla_{SHV}, bla_{GES}, $bla_{OXA\text{-}23}$
PA2	Tracheal aspirate		$bla_{CTX\text{-}M}$, bla_{SHV}, bla_{GES}, $bla_{OXA\text{-}23}$
PA3^{+}	Tracheal aspirate		$bla_{CTX\text{-}M}$, bla_{SHV}, bla_{GES}, $bla_{OXA\text{-}23}$
PA4	Tracheal aspirate		$bla_{CTX\text{-}M}$, bla_{SHV}, bla_{GES}, $bla_{OXA\text{-}23}$
PA5^{+}	Bronchial aspirate		$bla_{CTX\text{-}M}$, $bla_{KPC\text{-}2}$, bla_{GES}, $bla_{OXA\text{-}23}$
PA6^{+}	Blood		$bla_{CTX\text{-}M}$, bla_{SHV}, bla_{GES}, $bla_{OXA\text{-}23}$
PA7	Pus/wound		$bla_{CTX\text{-}M}$, bla_{SHV}, bla_{GES}, $bla_{OXA\text{-}23}$
PA8	Tracheal aspirate		$bla_{CTX\text{-}M}$, bla_{SHV}, $bla_{KPC\text{-}2}$, bla_{GES}, $bla_{OXA\text{-}23}$
PA9^{+}	Tracheal aspirate		$bla_{CTX\text{-}M}$, $bla_{KPC\text{-}2}$, bla_{GES}, $bla_{OXA\text{-}23}$
PA10	Tracheal aspirate		$bla_{CTX\text{-}M}$, bla_{SHV}, bla_{GES}, $bla_{OXA\text{-}23}$
PA11^{++}	Tracheal aspirate		$bla_{CTX\text{-}M}$, bla_{SHV}, $bla_{KPC\text{-}2}$, bla_{GES}, $bla_{OXA\text{-}23}$
PA12	Bronchial aspirate		$bla_{CTX\text{-}M}$, bla_{SHV}, bla_{GES}, $bla_{OXA\text{-}23}$
PA13	Blood		$bla_{CTX\text{-}M}$, bla_{SHV}, bla_{GES}, $bla_{OXA\text{-}23}$
PA14	Pus/wound		$bla_{CTX\text{-}M}$, bla_{SHV}, bla_{GES}
PA15^{+}	Tracheal aspirate		$bla_{CTX\text{-}M}$, bla_{SHV}, bla_{GES}, $bla_{OXA\text{-}23}$
PA16	Tracheal aspirate		$bla_{CTX\text{-}M}$, bla_{SHV}, bla_{GES}, $bla_{OXA\text{-}23}$
PA17	Tracheal aspirate		$bla_{CTX\text{-}M}$, bla_{SHV}, $bla_{KPC\text{-}2}$, bla_{GES}, $bla_{OXA\text{-}23}$
PA18	Tracheal aspirate		$bla_{CTX\text{-}M}$, bla_{SHV}, bla_{GES}
PA19^{+++}	Bronchial aspirate		$bla_{CTX\text{-}M}$, bla_{SHV}, bla_{GES} $bla_{SPM\text{-}1}$
PA20	Blood		$bla_{CTX\text{-}M}$, bla_{SHV}, bla_{GES}, $bla_{OXA\text{-}23}$
PA21	Pus/wound		$bla_{CTX\text{-}M}$, bla_{SHV}, bla_{GES}, $bla_{KPC\text{-}2}$
PA22	Tracheal aspirate		$bla_{CTX\text{-}M}$, bla_{SHV}, $bla_{KPC\text{-}2}$, bla_{GES}, $bla_{OXA\text{-}23}$

PA23	Tracheal aspirate		*bla*CTX-M, *bla*SHV, *bla*GES, *bla*OXA-23
PA24	Tracheal aspirate		*bla*CTX-M, *bla*SHV, *bla*GES, *bla*OXA-23
PA25	Tracheal aspirate		*bla*CTX-M, *bla*SHV, *bla*GES, *bla*OXA-23
PA26	Bronchial aspirate		*bla*CTX-M, *bla*SHV, *bla*GES, *bla*OXA-23
PA27	Blood		*bla*CTX-M, *bla*SHV, *bla*GES, *bla*NDM-1
PA28	Pus/wound		*bla*CTX-M, *bla*SHV, *bla*GES,
PA29^{+}	Tracheal aspirate		*bla*CTX-M, *bla*SHV, *bla*GES,
PA30^{+}	Tracheal aspirate		*bla*CTX-M, *bla*SHV, *bla*GES, *bla*OXA-23
PA31^{+}	Tracheal aspirate		*bla*CTX-M, *bla*SHV, *bla*GES,
PA32^{+++}	Tracheal aspirate		*bla*CTX-M, *bla*SHV, *bla*GES,
PA33	Bronchial aspirate		*bla*CTX-M, *bla*SHV, *bla*GES *bla*SPM-1
***Streptococcus* spp.**			
S1^{+}	Blood		
S2^{+}	Blood		*ermB*
S3	Blood		*ermB, tetM, pmr(A)*
S4	Blood		*tetM, mef(A), pmr(A),*
S5	Blood		*ermB, tetM, pmr(A),*
S6^{++}	Blood		*tetM, pmr(A)*
S7^{++}	Blood		*tetK, tetM, pmr(A)*
S8	Blood		*ermB, tetM, pmr(A)*
S9^{++}	Blood		*tetM*
S10^{+}	Blood		*ermB, tetM, pmr(A)*
S11	Blood		*tetM, pmr(A)*

$^{+}$: weak biofilm producer, $^{++}$: moderate biofilm producer, $^{+++}$: strong biofilm producer

Penicillin	Fluoroquinolone	Macrolide	Lipoglycopeptides	Sulphonamide	Aminoglycoside
Cephalosporin	Nitroheterocycle furans	Glycylcycline	Annamycin	Carbapenem	
